# Digging over that old ground: an Australian perspective of women’s experience of psychosocial assessment and depression screening in pregnancy and following birth

**DOI:** 10.1186/1472-6874-13-18

**Published:** 2013-04-09

**Authors:** Mellanie Rollans, Virginia Schmied, Lynn Kemp, Tanya Meade

**Affiliations:** 1 School of Nursing and Midwifery, University of Western Sydney, Sydney, Australia; 2 Centre for Health Equity Training Research and Evaluation part of the Centre for Primary Health Care and Equity, University of New South Wales, Sydney, Australia; 3 School of Social Sciences and Psychology, University of Western Sydney, Sydney, Australia

**Keywords:** Psychosocial assessment, Depression screening, Mental health, Women’s health, Postnatal depression, Domestic violence screening, Midwifery, Nursing

## Abstract

**Background:**

There is increasing recognition of the need to identify risk factors for poor mental health in pregnancy and following birth. In New South Wales, Australia, health policy mandates psychosocial assessment and depression screening for all women at the antenatal booking visit and at six to eight weeks after birth. Few studies have explored in-depth women’s experience of assessment and how disclosures of sensitive information are managed by midwives and nurses. This paper describes women’s experience of psychosocial assessment and depression screening examining the meaning they attribute to assessment and how this influences their response.

**Methods:**

This qualitative ethnographic study included 34 women who were observed antenatally in the clinic with 18 midwives and 20 of the same women who were observed during their interaction with 13 child and family health nurses after birth in the home or the clinic environment. An observational tool, 4D&4R, together with field notes was used to record observations and were analysed descriptively using frequencies. Women also participated in face to face interviews. Field note and interview data was analysed thematically and similarities and differences across different time points were identified.

**Results:**

Most participants reported that it was acceptable to them to be asked the psychosocial questions however they felt unprepared for the sensitive nature of the questions asked. Women with a history of trauma or loss were distressed by retelling their experiences. Five key themes emerged. Three themes; ’Unexpected: a bit out of the blue’, ‘Intrusive: very personal questions’ and ‘Uncomfortable: digging over that old ground’, describe the impact that assessment had on women. Women also emphasised that the approach taken by the midwife or nurse during assessment influenced their experience and in some cases what they reported. This is reflected in the themes titled: Approach: ’sensitivity and care’ and ’being watched’.

**Conclusions:**

The findings emphasise the need for health services to better prepare women for this assessment prior to and after birth. It is crucial that health professionals are educationally prepared for this work and receive ongoing training and support in order to always deliver care that is empathetic and sensitive to women who are disclosing personal information.

## Background

International research has identified the potential for significant short and longer term negative health and social outcomes for women and their infants of poor mental health in pregnancy and after birth [1-4]. Increasingly, policy makers and practitioners emphasise the importance of early identification and the need to offer services and appropriate treatment to women and their families [5]. As a consequence, psychosocial assessment and depression screening is now recommended as part of routine clinical practice of midwives and nurses working in Australia [6] and is increasingly being implemented internationally [7]. Assessment of psychosocial risk factors such as domestic violence, substance misuse, past history of abuse or mental health concerns, lack of support, lower socio-economic status and a stressful pregnancy [8,9] has become a key component of routine antenatal and postnatal care for Australian women in the state of New South Wales (NSW). The aim of the State policy known as ‘Supporting Families Early’(SFE) [5] is to identify women with known risk factors (see Table 1 for assessment domains and questions) and to provide women and their families with ‘appropriate information and additional appointments or referral’ [5] p.69. The assessment process includes screening for depressive symptoms using the Edinburgh Postnatal Depression Scale (EPDS), domestic violence screening and questions about drug use and previous or existing mental health issues (see Table 1). The SFE policy recommends that women be assessed as a minimum, at two points in time: antenatal (approximately 12–14 weeks gestation) at their hospital booking visit and again approximately two–four weeks after birth at the time of the routine health home visit or at the six–eight week baby check in the clinic setting. In the NSW public health system, the first of these assessments is undertaken by a midwife at the hospital booking visit for pregnancy care and the second by the child and family health nurse (CFHN) who, similar to the health visitor in the United Kingdom, provide preventative health for children and families from birth to five years of age.

**Table 1:** **Psychosocial assessment domains and questions [**[5]**]**

**Variables (Risk Factors)**	**Suggested format for psychosocial assessment questions**
I. Lack of support	1. Will you be able to get practical support with your baby?
	2. Do you have someone you are able to talk to about your feelings or worries?
II. Recent major stressors in the last 12 months.	3. Have you had any major stressors, changes or losses recently (i.e., in the last 12 months) such as, financial problems, someone close to you dying, or any other serious worries?
III. Low self-esteem (including lack of self-confidence, high anxiety and perfectionist traits)	4. Generally, do you consider yourself a confident person?
	5. Does it worry you a lot if things get messy or out of place?
IV. History of anxiety, depression or other mental health problems	6a. Have you ever felt anxious, miserable, worried or depressed for more than a couple of weeks?
	6b. If so, did it seriously interfere with your work and your relationships with friends and family?
	7. Are you currently receiving, or have you in the past received treatment for any emotional problems?
V. Couple’s relationship problems or dysfunction (if applicable)	8. How would you describe your relationship with your partner?
	9. a) Antenatal: What do you think your relationship will be like after the birth?
	b) Postnatal (in Community Health Setting): Has your relationship changed since having the baby?
VI. Adverse childhood experiences	10. Now that you are having a child of your own, you may think more about your own childhood and what it was like. As a child were you hurt or abused in any way (physically, emotionally, sexually)?
VII. Domestic violence (DV) questions must be asked only when the woman can be interviewed away from partner or family member over the age of 3 years. Staff must undergo training in screening for domestic violence before administering questions.	11. Within the last year have you been hit, slapped, or hurt in other ways by your partner or ex-partner?
	12. Are you frightened of your partner or ex-partner? (If the response to questions 11 and 12 is “No” then offer the DV information card and omit questions 13–18)
	13. Are you safe here at home?/to go home when you leave here?
	14. Has your child/children been hurt or witnessed violence?
	15. Who is/are your children with now?
	16. Are they safe?
	17. Are you worried about your child/children’s safety?
	18. Would you like assistance with this?
Opportunity to disclose further	19. Are there any other issues or worries you would like to mention?

Studies in Australia and overseas report that most women find routine antenatal psychosocial assessment and depression screening to be acceptable [10-14] offering them an opportunity to discuss sensitive issues [11-13]. In telephone interviews with a large sample of community women, Leigh and Milgrom [14] found 100% acceptability of screening for depressive symptoms by midwives using the EPDS in pregnancy. Buist et al. [15] found a similarly high level of comfort with depression screening (85%) however, they reported that women with an EPDS ≥ 13 were more likely to find the screening process uncomfortable. Matthey et al. [16] also conducted telephone interviews before after birth to ascertain acceptability of psychosocial assessment and depression screening. They found that 65% of English-speaking women thought the psychosocial questions were acceptable, with the remainder qualifying their response indicating that they or other women may not be happy to answer certain questions such as those related to domestic violence and were uncertain as to why some questions about their childhood were relevant. One fifth (19%) of women were ambivalent or negative about the questions [16].

Conversely, concerns have been raised both nationally [17] and internationally [18,19] about the use of assessment tools particularly with vulnerable women as they may feel judged or victimized by the questioning and may deny problems such as domestic violence or their own negative childhood experiences, paradoxically placing them at greater risk of reduced access to supportive services [17-19]. Few studies have investigated the style and approach that midwives and nurses take to conducting the assessment or how women respond to the questions and make meaning of this experience.

This paper reports the findings of one part of a larger ethnographic study that sought to describe the process and the impact of psychosocial assessment and depression screening. The main focus of the study was; the approach midwives and nurses take to the assessment process and to report the midwives', nurses' and women’s experiences. This paper describes women’s experience of psychosocial assessment and depression screening examining the meaning they attribute to assessment and how this influences their response. The experiences of midwives and nurses have been reported separately [20].

## Method

### Study design

This ethnographic study was conducted in NSW, Australia between September 2010 and October 2011. Data were collected through observations of the first antenatal visit at the hospital (the booking visit) and the first visit by nurses in the home or at the clinic 6 weeks after birth. Interviews were conducted following the observations with all participating women. The opportunity to both observe the women as they interacted with Midwife or CFHN and how they felt about these questions is central to this study. Directly observing interactions, between women and midwives/CFHN assisted to understand the context of women’s experience. The subsequent interviews added depth to the observational data by asking women how they felt about being asked the assessment questions and whether this had any longer term impact on their relationship with the services. Informed written consent for participation in the study was obtained from participants. Ethics approval for the study was obtained from the Human Research Ethics Committees at both study sites and from the University of Western Sydney.

### Study setting

Women were recruited from two maternity units. Both sites (A and B) provide publically funded maternity care to over 3,000 births per year and are located in areas with a diverse multicultural population. At both sites assessment and screening processes had been established for over five years and a coordinated response /pathway was in place for women identified with potential risk factors for poor mental health. The process of assessment differed slightly at each site. The length of time allocated to conduct the visit at site A was one hour and at site B, one and a half hours; and at site B partners were able to attend for some of the visit and at site A, partners were unable to attend the booking visit. At site A, CFHN undertook the assessment at the first home visit and at Site B nurses were instructed to undertake the assessment at the six week visit when the mother came to the clinic.

### Participants and recruitment

#### Women

Potential participants were informed about the study via information leaflets included in a package mailed to women by the hospital prior to their first appointment (booking visit). The first author was available on a regular basis in the waiting area of the antenatal clinic and approached women attending their booking visit to provide details about the study and invite them to participate. A total of 60 women were approached antenatally and 34 agreed to be observed during the booking visit with the midwife and at the first appointment with the CFHN services. They were also asked if they agreed to participate in a face to face or telephone interview two to four weeks following each observation.

Women were excluded from the study if they spoke insufficient English to participate in a face-to-face interview without an interpreter. This was necessary as the study focused on the interactions between professionals and women and the interaction may be altered if an interpreter is present. These exclusion criteria, however, did not limit participation of women from non English speaking backgrounds.

We anticipated that 30 women (15 in site A and 15 in site B) would provide sufficient rich qualitative data across three data sets: observations, field notes and interviews from all participants. Thirty four women were recruited to the study. Guest et al. (2006) notes that for qualitative research that aims to understand patterns and commonalities in experience and perception, 12 participants will provide sufficient data. At each of the two sites a minimum of 15 women were targeted to provide a reasonable representation of the women across both sites. The authors did not specify the background of women, nor limit the study to first time mothers, as we wanted to capture the experiences of the general population of women who are using these services and exposed to routine psychosocial assessment.

On average participants were 30 years of age, over half (20 out of 34) were born in a country other than Australia. Five of these 20 women were born in English speaking countries such as Ireland, United Kingdom and 15 women were born in non- English speaking countries such as Egypt, Laos, India and China. Eleven women spoke a language other than English. Eighteen of the 34 women were having their first baby, however, 10 of these women had previous pregnancies but had no living children due to miscarriage or termination of pregnancy. The participants were well educated with 30 of the women having tertiary qualifications and all participating women were either married or living with their partner who was the father of the baby.

Initially all 34 women agreed to being observed at both time points, however in the postnatal period only 20 of the 34 were observed. The remaining 14 women were not observed due to varying circumstances such as relocating out of the area where ethics approval had been obtained for observations (n = 5), withdrew from the study (n = 4), challenges involving co-ordinating visits with CFHN and the women (n = 2), did not attend their scheduled appointment (n = 2) or refused postnatal visit from the CFHN (n = 1). However, 9 out of the 14 remaining women agreed to participate in follow-up interviews even though observations were not conducted.

#### Midwives and CFHN

Sixteen midwives, two student midwives and 13 CFHNs participated in the study. The midwives and CFHNs were informed about the study through a series of researcher led discussions in staff meetings conducted at each site. Interested midwives/CFHNs completed consent forms and returned these to the researcher. Opportunity to participate in this study was offered to all midwives, working in the antenatal clinic and CFHNs who provided Universal Health Home Visit (UHHV) or clinic services, where psychosocial assessment was conducted. Student midwives were also included as participants as they were conducting psychosocial assessment and depression screening at the antenatal booking visits. The researcher then attended the antenatal clinic on days that the consenting midwives were working in order to recruit women and observe the interactions. Women who were observed antenatally and agreed to participate in the postnatal observations were matched with a consenting CFHN conducting the home visit or the 6 week visit, at which time the researcher (MR) was present to observe the interaction.

The average years of experience of the 16 midwives was five years and 12 of these midwives had worked an average of three years in the antenatal clinic. The professional experience of the CFHN ranged from less than 1 year to over 20 years. Eight CFHN had greater than five years experience. One CFHN worked on a casual basis, the remaining CFHN were employed in a permanent capacity either part or full-time.

Note: Unless specifically referring to midwives or CFHN the term midwife/CFHN refers to both midwives and CFHN.

### Data collection

#### Observations

All data were collected by the researcher (first author MR) who is an experienced clinician in this area. The researcher (MR) observed all interactions between women and midwife/CFHN at two points in time, once antenatally and once postnatally and conducted face to face interviews. During data collection, over an 18 month period, the researcher (MR) was provided with training, support and regular supervision to ensure the quality of data collection and ‘reliability’ or consistency in recording the observations. The researcher (MR) led the development and pilot tested an observational tool (4D&4R) [21]. Due to the researchers' (MR) familiarity with the tool, data was recorded in a consistent manner, in all settings. Nutley et al. [22] reports that consistency amongst the usage of tools aids reliability of data sets. During supervision meetings with MR, observation tool data and field notes were reviewed by the co-researchers.

Nutley et al. [22] note that careful preparation of an observational tool can help plan how data will be recorded and can identify focal points during observations that are central to the study’s objectives [23]. The development of the observational tool (4D&4R) used in this study included (i) consideration of the study requirements in relation to the aims of the study and the study’s context; (ii) previous research in this similar context; and (iii) the authors collective and complementary cross-disciplinary knowledge and experience relevant to the study’s context, content or the methodological framework. A literature review was conducted to identify aspects of communication processes that were to be observed; the means by which previous studies recorded observational data and if a tool existed that could be applied to this study [21]. A tool consistent with this study’s objectives was not identified, therefore, an observation tool (4D&4R) was developed for the study [21].

The 4Ds (introDuce, Deliver, Deal and Debrief) were designed to record details about the approach taken by both midwives and nurses to the psychosocial assessment and screening. The 4Rs (React, Respond, Real experience and Reflect) were designed to observe and record details of the woman’s response, including aspects such as how the woman reacted to being asked sensitive and intimate questions, what physical indicators denoted a reaction (i.e. flushed face, smiling, frowning etc.); how the woman responded, was she open and talkative in her response or did she withdraw from responding using monosyllabic responses or chose to not verbally respond at all; what was the real experience or how congruent did the woman appear (e.g. tearful at discussing traumatic event however denying that she was distressed) and was the woman observed to reflect on the questions being asked (i.e. did she ask to clarify one of the questions or did she raise her response to a previous question at some other point during the interaction) (a more detail discussed on the observation tool is reported in [21].

Observations occurred with 15 women and seven midwives (including one student midwife) at site A and with 19 women and 11 midwives (including one student midwife) at site B. The postnatal observations of interactions between CFHN and 11 women at site A took place at the home visit conducted by the nurse two to four weeks after birth. At site B, nine women were observed in the health centre where the assessment was conducted by the nurse at six weeks after birth. This difference in time points of data collection was in response to differing implementation of the Safe Start policy [5] across the two participating sites and was unavoidable.

#### Field notes

Detailed field notes were used with the observational tool to document verbatim the conversation between the woman and the midwife/CFHN during psychosocial assessment and screening. Notations were also made to elaborate on the non verbal communication observed. These interactions were not audio recorded as this may have been intrusive or interfere in the interactions [24], especially where sensitive information is revealed. Briggs [25] indicates the important work that midwives and nurses do to engage women and families at the points in time when observations were conducted. The authors determined that using non-technological approaches to record observational data was less intrusive and more sensitive to these types of interactions.

#### Interviews

Semi-structured face-to-face interviews were conducted with 31 women in the antenatal period. These comprised 23 face-to-face interviews and eight telephone interviews within 3–4 weeks following the observation of the booking visit. Interviews were conducted at the maternity unit when the women returned for her next appointment. The telephone interviews occurred two to four weeks after the observation at a time convenient for the woman. Following birth, 29 women agreed to an interview approximately two–four weeks after the observation; 19 of these were conducted on the phone and 10 were face-to-face at a time suitable for the woman and typically in her home. These interviews comprised a series of open-ended questions to elicit information about women’s experiences with the assessment process (see Table 2). The interviews took approximately 15–40 minutes and with permission all interviews were digitally recorded. Interviews were transcribed verbatim with all identifying material removed.

**Table 2:** Interview questions

**Questions from researcher to woman in private interview**
1. Overall how did you find being asked the questions related to your personal situation? (the psychosocial assessment questions)
2. Were there any questions that you felt were helpful?
3. Were there any questions you felt were uncomfortable or more difficult to answer?
4. Was there anything particular your midwife or nurse did that made you feel comfortable?
5. Do you have any thoughts about what could be done differently to help other women being asked these questions?

To ensure credibility and transferability of the findings, women in this study voluntarily agreed to participate and were recruited within a setting where assessment and screening is conducted, the antenatal clinic. Women participants were from diverse backgrounds and represent the broader population of women who attend antenatal and postnatal clinic areas, where assessment and screening is conducted. The researcher (MR) spent time in the clinic area gathering data, developing familiarity with the context and the environment. The same methods of data collection were applied to all settings and with all participants by the same researcher (MR), i.e. the use of field notes and the 4D&4R observation tool to record interactions and face to face interviews.

#### Data analysis

Textual data from the field notes of observations and interview transcripts were analysed thematically [26]. The observational tool (4D&4R) data were analysed descriptively using frequencies and proportions [21]. All the data including data from the observation tool (4D&4R), field notes and interviews with the women, both antenatal and postnatal, were analysed simultaneously and involved two stages of analysis. Firstly; the iterative process of thematic analysis was conducted to identify the themes and subthemes across all textual data. Secondly; the emerging themes were examined to determine if they were consistent or not across the women’s experience at both points in time and in the context where assessment was conducted, home versus the clinic. Analysis involved multiple readings and re-readings of the observational data and listening to the recordings of interviews, this was conducted by author MR. Codes were identified that described the process of psychosocial assessment with illustrations of interaction data and the women’s response to the assessment. This was an iterative approach which involved all researchers discussing the concepts, themes and relationships during analysis. Emerging themes and the accompanying data were reviewed by the co-authors to ensure reliability of the coding. Concepts and themes were constantly compared with other themes and refined [27]. This process resulted in identification of key themes [28]. These themes are presented in phrases that, where appropriate, use the verbatim language of the participants. The descriptive data obtained from the 4D&4R data provided frequencies of women’s responses consistent with the emerging themes of women’s experience.

## Results

Five key themes emerged from this analysis. The first three themes titled ‘Unexpected – a bit out of the blue’, ‘Intrusive - very personal questions’ and ‘Uncomfortable - digging over that old ground’ describe the impact that psychosocial assessment and depression screening had on women. The lack of preparation or surprise that women experienced in relation to being asked sensitive questions that may bring up past, difficult experiences can be modified by the approach that the midwife/CFHN takes to asking the questions. This is reflected in final two themes titled: Approach: ‘sensitivity and care’ and ‘being watched’.

In addition to reporting the five major themes that emerged across the three data sets, the results reflect the longitudinal aspect of the study and aspects of the women’s ‘journey’ across the two time periods when assessment occurred. Exemplars from two women are included to illustrate where women’s experience and their responses either antenatally or postnatally, may have differed. These exemplars occur in the themes sensitivity and care and uncomfortable: ‘digging over that old ground’.

### Unexpected – ‘a bit out of the blue’

Analysis of the observation tool (4D&4R) and interview transcript data indicate that when the midwife/CFHN first introduced the psychosocial assessment, whether that was at the start of the visit or well into the interaction, women appeared ‘*surprised* ‘or ‘*perplexed*’. This was particularly so for women at the antenatal booking visit as this was often the first time they were asked these questions, but was also observed with some women having the home visit or clinic appointment after birth. Most of the women expressed in interview that they didn’t ‘*…expect…*’ the level of personal detail required to be shared at the visits and felt ‘*…surprised…*’ by the personal nature and sensitivity of the questions, it came ‘*a bit out of the blue’* (W9). However, they remained open to answering the questions stating at interview they ‘*understood*’ why the questions were being asked;

‘*I think the questions that they ask are sometimes very personal, but on the other side I understand why they have logic behind the asking. They want to find out about the woman so they can help her. Sometimes there were a few questions that were very personal, like about your sexual history, that a person doesn’t like sharing the first visit.’* (W18).

Women stated they were expecting the focus to be on either the physical progress of their pregnancy or the health and development of their baby rather than on their own emotional health and well-being, *‘It seemed to be more about me than the baby’* (W11); ‘*I felt it was more about me and it was more about my mental state, that sort of stuff…it was really more about how are you feeling? How are you coping?*’ (W7). Almost half, (25 out of 54) of the women observed either antenatally or postnatally, demonstrated a physical reaction when asked the psychosocial assessment questions. For example, some women showed a surprised expression (raising of the eyebrows, crinkling of the forehead skin), increased eye movement, a sudden turn of the head to face the midwife/CFHN and shifting in the seat. However, after this immediate or initial reaction, two thirds of the women, (38 out of 54) responded to the questions in an open and talkative way;

(FN5) Midwife was observed to ask the woman about asking questions related to W5 psychosocial history:

M2 - …there is also a section about you and I’ll ask you some questions about your social and emotional wellbeing is that okay?

W5 – (turn her head and looks sharply at midwife) what do you mean, what sort of questions?

M2 – they’re just some questions to see how you’re coping and feeling stuff like that

W5 – Oh okay then (sits back in chair looks more relaxed and smiling)

Some women questioned the relevance of the psychosocial assessment questions ‘*She didn’t check the baby’s (fetal) heart rate it just seemed to be more about me, I’m not even sure why those other questions* (psychosocial assessment questions) *were relevant’* (W19).

Women were also surprised that the antenatal and postnatal visits took much longer than they expected. They attributed this length of time to be about the paperwork or the amount of questions that were asked;

‘*I was surprised it took so long, I left mum in the car waiting, I didn’t bring a bottle or a change of clothes with me or anything, I just didn’t think it would take so long’* (W7) or ‘*was basically paperwork, we did lots of paperwork rather than checkups’* (W31).

Women’s level of preparedness for the psychosocial assessment questions seemed to impact on their experience. Some of the women experienced discomfort stating that antenatally they were *’…not feeling adequately prepared…*’ (W4).

W16 – I think they could have told me what they were going to ask before I even arrived for my appointment. I had no idea that was what was coming.

However, women who had a recent previous pregnancy and birth felt they were more prepared and appeared more relaxed;

‘*It was actually quite good. I actually quite enjoyed it. The first time you didn’t know what to expect and some of the questions that they asked I thought were a bit surprising but the second time round it was like it was nothing. It was just a conversation; they just needed to know information’* (W4).

During pregnancy and following the birth, it was evident that women expected the midwife or nurse was going to provide answers to their questions or advise them about caring for their baby, for example;

W6 – When I went for my first visit with the midwife I was like, right this is what we need to know and I just felt confident that the midwife would know exactly how to answer my questions

*W1* - *I knew that the nurse would come here to my home and help me to solve some of the problems with my baby’.*

### Intrusive: very personal questions

During interviews women stated that they understood why the midwife/CFHN was asking the questions and they believed that it was ‘.*..important…*’ and that they‘*…should be done…*’ (W16). When women had a positive experience disclosing a recent difficult personal experience, they were more likely to develop a sense of trust in the midwife that they ‘*…could tell my story to anyone’* (W12). However, women also described them as ‘*… very personal questions…’* and this evoked some discomfort at times and may have influenced whether they shared their story with the midwife/CFHN. Whilst most women explained that they responded honestly to the questions, stating they ‘*… had nothing to hide…*’ (W21), some women did say that if they were experiencing distress they may not have disclosed this to the midwife/CFHN and would simply say ‘*…no…*’ (W26). Due to the personal nature of the questions women reflected on whether they would discuss personal concerns with midwife/CFHN;

*They ask very personal questions about your social circumstances and your relationships and things like that, which I have no problem answering but I can see how some people would if they had a problem it would be very hard to bring up like domestic violence or abuse or something like that* (W26).

The observation data demonstrated support for women’s level of openness and honesty. In almost all of the observation (53 out of 54) women responded to questions and some took some time to reflect on the answer. On one occasion when a woman was tearful she continued to exclaim that she was ‘*okay…no I’m alright*’ (W20) and declined to discuss any concerns with the midwife/CFHN. In 5 out of the 54 observations women were offered time to reflect on the questions they had been asked. In these instances women pondered for a moment to reflect on what they had been asked ‘*I guess I’ve never thought about it really but yes I guess I was a bit depressed when I was a teenager*’ (W30).

There appeared to be particular questions that caused women some discomfort. Questions about domestic violence were described as ‘*…strange…*’ and ‘…*funny…*’ One woman described her discomfort as ‘…*feeling guilty…*’ (W11) about being asked the domestic violence questions as;

*‘ I kind of likened it to you know when you come through customs and even though you know you’ve got nothing; you feel guilty because you now that customs people are there and they might think you’ve got something in your bags, even though you know you haven’t…it was a bit like that feeling’* (W11).

Similarly, some questions that were not deemed to be part of the psychosocial assessment were viewed as ‘*personal questions*’ by the women and provoked discomfort. For example, questions about previous pregnancies such as terminations or stillbirth ‘*I was really hoping it wouldn’t come up… but when it does it’s like all this emotion just exploding out of me…*’ (W12).

### Uncomfortable: ‘digging over that old ground’

Women who did disclose a difficult current or past life event or experience in response to a question described the impact of this in varying ways. One woman described this as ‘*digging over that old ground’* (W9). For others, talking about previous traumatic histories was *‘…daunting…’*, raising fears that women may be ‘*…pushed back…’* into reliving previous trauma. One woman stated, ‘*when those words come up again (postnatal depression) you don’t want to be pushed back, like when the help is offered it’s wonderful but I felt, no I’ll be able to cope this time.’* (W12) The retelling of a distressing experience may impact in a negative way on a woman’s mood;

‘*It did feel like it brought up a lot of feelings for me, like the anxiety I had when I was developing postnatal depression with my first child, it was all there, I could feel it again’* (W12).

Another woman also talked about her distress when asked to talk about a recent still birth. This was the first time she had returned to the hospital following a recent stillbirth;

*it’s difficult to reply to all those questions with all my background and all my past. It makes me so stressed, when I have to go through all the questions. That’s my personal* (feeling)*. Maybe if people have a good past they’ll enjoy it* (W31).

Discussing previous trauma may not appear relevant for women at the present time of the visit ‘…*it’s not really affecting me now…my main concern is getting through the pregnancy, not worrying about my past stuff*.*’* (W21). Women also described how they had to think carefully about how to respond to a sensitive question;

about suicidal and depressed – yeah, I was thinking actually how I put this?… because it’s actually hard to know how to say it, so you go, well, ‘how do I say this, yes I have had a plan to end my life cause I just didn’t see a way out anymore (W18).

Women who did disclose previous trauma mostly felt the nurse or midwife responded appropriately, however, they suggested that the midwife/CFHN should review previous notes so they ‘*don't have to go real deep, they can just open my file instead*’ (W31) or;

‘*Why don’t they take the extra time just to read over and if they have any more questions about it then they can ask. If it’s already there then why bother… it is really frustrating’* (W4).

At times, it was the response of the midwife or nurse to a woman’s disclosure that caused them the most distress. One woman (W28) talked of her experience when she disclosed a previous history of anxiety, although this had not been formally diagnosis or treated. In this case, the midwife (M16) documented this information as a history of previous mental health problems on her medical record card. The woman saw this documented and in the interview she stated;

*I didn’t know that was written on the card, when I saw that there I was surprised because I don’t feel like I’m depressed or have anxiety. So I think that process made me anxious. Because now they* (other midwife/CFHN) *ask me a lot about it and I am looking at it as a kind of an issue so it’s creating like a dirty mark against my name* (W28).

Some women who described a negative experience of psychosocial assessment indicated that they would inform other women and discourage other women from disclosing personal information;

*I don’t want her* (sister) *to go through the whole thing, I don’t feel the need for her to bring it up, and I don’t want her to go through her whole pregnancy having to see someone about her problems. So I told her about some of the stuff in regards to some of the questions they ask about your husband, whether or not he beats you up and I told her so she knows what will happen if she says anything* (W4).

*It’s funny because when you talk to girlfriends who’ve had children, you hear everyone’s different experiences and they say ‘it’s when they ask the questions it’s like, you know, it’s crazy* (W25).

In some instances women’s comfort regarding disclosure of previous negative life events to the midwife or nurse differed across the two points in time (antenatal or postnatal). The exemplar in Table 3 illustrates how W17s negative experience at the antenatal psychosocial assessment influenced her decision not to use postnatal services. At the antenatal booking visit, W17 disclosed a previous experience of domestic violence which occurred more than two years previously with an ex-husband who lives abroad. This history of domestic violence was incorrectly reported by the midwife in the medical records as occurring currently. Following birth, W17 was visited by a social worker who interviewed her about the safety of her current relationship with her husband. This was particularly distressing and intrusive for W17 and she subsequently refused ongoing services.

**Table 3:** W17s experience of psychosocial assessment across time

**Antenatal interaction with midwife**	**Antenatal interview with W17**
*Student Midwife 1 (S1) – In the last 12 months have you been hit, slapped or hurt by your partner or ex-partner?*	*Researcher* [MR] *– What did you think of being asked the questions?*
*W17 – No*	*W17 – I was surprised, I was really upset, I think they just check up the baby but too much question, I’m shy*
*S1 – Are you frightened or scared of your husband or ex-husband?*	
*W17 – My ex husband*	
*S1 –* [S1 explores] *Do you have much contact with him*	
*W17 – A social worker calls him and calls me*	
*S1 – When you say you are scared of him is that now?*	
*W17 – I’m not scared of him,* [loudly] *long time ago, for past 2 years.*	
*FN17 – Researcher* [MR] *observes S1 recording a positive response to domestic violence into W17 medical records*	
**Postnatal interaction with researcher**	**Postnatal response to negative antenatal experience**
*FN17 – Researcher* [MR] *contacted W17 via telephone to confirm postnatal observation of interaction between W17 and CFHN. W17discussed that she had refused the routinely offered health home visit by the CFHN. W17described the inaccurate recording of a positive response to domestic violence question by the midwife; resulting in W17 being seen by a social worker within 24 hours of delivery. W17 was asked further questions as to the nature of her relationship with her current husband.*	*W17 - They ask all those personal questions and they got it wrong.... I don’t want them to come to my house; No…I don’t want to see anyone anymore*

There were women who were dissatisfied with the health service. For example, some women were informed by the midwife/CFHN that they would be referred to services and then they did not subsequently receive any follow up. ‘*I waited and waited for her to call for two weeks… she said the social worker would call…but nobody called’* (W18). Women suggested that a collaborative approach to recording the information gathered would demonstrate a level of respect and involve them in their care more productively ‘*maybe if she* (the midwife) *had asked me what I wanted put down on my card I would have felt she was helping me and being more considerate of me’* (W28). However, the types of support that midwife/CFHN provided such as information and contact numbers was positively received ‘*The information she gave me I felt quite helpful and the telephone numbers so I know where to go’* (W5).

### Women’s perception of midwife/CFHN style and approach

#### Approach: sensitivity and care

It was evident in this study that how women perceived the midwife/CFHN style or approach influenced their level of comfort with the clinical encounter in general and in particular with the psychosocial assessment and depression screening. The majority of the women described the midwife/CFHN as being ‘friendly’, ‘warm’ and ‘caring’ and they believed that the professionals were ‘*sensitive*’ to their needs: ‘*I felt she was very friendly and quite professional in the way she talked to me, I felt very relaxed.’* (W1) or ‘*I found it a quite positive experience overall, I thought her approach was sensitive and caring, it was friendly’* (W24). Some women indicated that asking the questions implied a ‘…*sense of caring…*’ (W5) on the part of the midwife/CFHN.

Women were appreciative when the midwife or CFHN was sensitive and caring. For example, in the first interaction in Table 4, Midwife (M9) demonstrated empathy and validated the woman’s experience by acknowledging her difficult situation. Similarly when W11 disclosed that she had been experiencing emotional distress; she stated that she felt supported by the response from CFHN6 inviting her to discuss further (see Table 4).

**Table 4:** Examples of sensitive interactions

**Woman/Midwife interaction**	**Woman/ CFHN interaction**
*M9 – Have you had any major stressors in the past 12 months?*	*W11 – No I think I’ve coped fairly well, but is it normal to feel a bit emotional like during breastfeeding, it’s a bit like premenstrual… I have had a bit going on surprisingly?*
*W30 – Only the miscarriage(looking down into hands clasped in the woman’s lap)*	
*M9 – That’s a toughy… that would have been hard, I’m sorry about that (midwife turns to look at woman and smiles gently)*	*CFHN 6 – yep, yep definitely with all the hormones, but has there been anything else that has been troubling you that you’d like to talk about?*
*W30 – (woman looks up and turns to face the midwife) Yes it was really hard*	

Women also described the midwife/CFHN as helpful in terms of problem solving or assisting them to accept their pregnancy and the impact this may have on their psychosocial wellbeing. For example;

M5 – How are you feeling about being pregnant?

*W10 – yeah ok* (ambivalently)*, he’s* (turns to look at partner) *so excited, I’m kind of getting used to it*

M5 – Okay, I remember feeling like that too when I first got pregnant, what sort of things would help you to feel more comfortable about the pregnancy?

The women who came from non-English speaking backgrounds emphasised the importance of the non verbal communication from professionals commenting for example, on the midwife/CFHN facial expressions as an indicator of friendliness ‘*she always smiles and* (is) *very gentle’* (W14) or ‘*She make me like not scared because she smile a lot, her smiling and the way she spoke was really helpful’* (W3). They also expressed an ease of communication when the midwife/CFHN ‘*spoke slow to me’* (W17) and *‘explained the questions, even if I didn’t understand first time round’* (W2).

In contrast however, there were interactions where women did not receive an empathetic or sensitive response from their midwife or CFHN, as illustrated in the following interaction. For example, one woman (W4) expressed that she would prefer the midwife to refer to her medical records where she had previously disclosed trauma when asked during her first pregnancy;

M3 – so as a child were you hurt or abused in any way either physically, sexually or emotionally?

W4 – Yes

M3 –Did you want to tell me about that?

W4 – Isn’t it in my file from last time?

M3 – No… Well I haven’t read it… you can tell me about it now?

FN - Woman described her experience reluctantly

There were instances where women reported different experiences of sensitivity and care in interactions with the midwife or the nurse across the two time points (antenatal and postnatal). The exemplar in Table 5 demonstrates W12s' experience of M2s' sensitive and caring approach during her disclosure of a recent termination of pregnancy. This same woman (W12) however, was distressed by the response from the CFHN7 in the postnatal assessment where she disclosed that she felt traumatic by her caesarean birth. During interview with the researcher (MR), W12 stated that the nurse lacked sensitivity and caring.

**Table 5:** W12 experience of assessment across time

**Antenatal interaction W12 with M2**	**Antenatal interview with W12 – sensitivity and care**
*M2 – Any past pregnancies…?*	*Researcher* [MR] *– what did you think of being asked the questions?*
*FN12 -* woman observed to be distressed by this questions and cries	
*M2 – That’s alright, you’re still upset, you don’t have to talk about it*	*W12 – I knew the minute the question was asked* [previous pregnancy],
*W12 – I was dreading this coming up*	*I felt this from the inside just explode out of me. She* [M2] *was brilliant,*
*M2 – sorry, some of the questions are quite personal*	*I felt supported, not judged and walked away feeling I could tell my story to anyone*
**Postnatal interaction W12 with CFHN7**	**Postnatal interview with W12 – lack of sensitivity and care**
*CFHN7 – You have a few responses here, how have you been feeling?*[note here the nurse is intending to explore the high score on the EPDS]	*Researcher* [MR] - *How did you think the clinic visit went overall?*
*W12 – There’s definitely a difference especially the way I feel physically*	*W12 – I started to get upset after that meeting, I just assumed that anyone who deals with mothers and babies is just well – they are really caring and nurturing but I didn’t feel that. I felt like it was a reflection on me, that I was bad. I felt judged*
*CFHN7 – Because you are feeling…?*	*Researcher* [MR] – *Is there anything the nurse could have done differently?*
*W12 – Like I’ve been attacked with a machete*	*W12 - Maybe if they talked to women, or explored it. I felt like I’d had an operation, I was really scared. Maybe theycould be empathetic and sensitiveto people’s emotions because a lot of people experience things differently.*
*CFHN7 –* [surprised] *what? Because of the caesarean? I’ve had those…*	
*W12 – I have found it hard to relax since*	
*CFHN7 – well you’re just going to have to learn…*	

### Approach: being watched

Whilst most women were approached sensitively, some women talked about feeling as though they were being watched during the home visit by the CFHN. This was particularly reported by women for example when the CFHN commented about aspects of the home environment. This was reflected in the following interaction in the home between a nurse and a woman,

*CFHN10 – You’ll need to get a gate for here at the bottom of these stairs…and what’s that cheeping sound… you’ll need a new battery for that fire alarm…do you mind if I go outside and take a look....*(walks to the backdoor) *no buckets of water laying around anywhere?*

*W25 – We’re planning to do this* (install gates at the bottom of the stairs)*....we just waiting till the baby is a bit older*

CFHN10 – You need to think about this as a safety thing

In the interview following this observation, this woman stated that she felt uncomfortable with the nurse describing the interaction as ‘*rude’* and *‘intrusive’*.

W25 - That was a bit uncomfortable for me, when she’s checking everything…I was surprised she wanted to see how I lived…it was a bit strange, if I’d gone to the clinic she’d never have known any of these things. That was a little bit rude I thought. I preferred not to see them (CFHN) again.

Another woman described feeling *‘very upset’* and *‘guilty’* following the home visit. She described that on entry to her home; the nurse began to survey the kitchen area as though assessing the level of cleanliness and then proceeded to involve the woman’s in-laws in a discussion about breastfeeding routines. The woman was distressed by this because at the time she was experiencing some conflict about breastfeeding, she wanted to breastfeed and her parents’ in-law were discouraging her;

*W11 – It was a difficult thing* (breastfeeding) *at the time and she* (nurse) *made a comment about my breastfeeding to the in-laws saying ‘if you don’t get her feeding properly then we’ll have to blah blah blah’ and this just escalated things because the family got even more concerned.*

The women’s experience of being watched was only reported and observed in the home environment after birth and not the clinic setting. Women who were assessed in the clinic before and after birth did not report statements about being watched.

## Discussion

This paper aimed to describe women’s experience of psychosocial assessment and depression screening examining the meaning they attribute to assessment and how this influences their response. There are few studies that have examined in depth women’s experience of psychosocial assessment and depression screening and the approach taken in this study is unique. This study observed the interactions between midwives, nurses and women during psychosocial assessments and explored, through interviews the experiences of the same group of women during assessment before and after birth. The study found that while most women appeared to be accepting of the questions, they were taken by surprise and felt unprepared for this part of the clinical encounter with the midwife/CFHN. Overall, participants described the assessment positively believing that it demonstrated care on the part of the professional and the health service and provided them with an opportunity to talk further about any issues they may have if they wished. They also believed there was value in asking women these questions. However, women who answered positively to the psychosocial questions reported mixed feelings, with some finding the questions daunting and intrusive. One of the key factors that influenced whether the woman had a positive or negative experience at this time was the skill of the midwife or nurse and their capacity to respond sensitively to the woman.

Research conducted in Australia and elsewhere into the acceptability of psychosocial assessment by Matthey et al. and Rowe et al. [16,29] and depression screening by Leigh and Milgrom and Buist et al. [14,15] similarly reports women’s general acceptance of the assessment. In contrast to these previous studies based on data collected through structured surveys or telephone interviews, when women were observed in interactions with midwives and nurses in this study, some of the women were visibly uncomfortable, shifting in their seat or looked slightly flushed and others stated in interviews with a known researcher that they found some of the questions uncomfortable and did not want to *‘dig up the past’*. This suggests that when this experience is explored in an in-depth way and over time a more complex picture of positive and negative experiences emerges.

Participants described being unprepared and surprised by the length of time the assessment took, the number of questions and the sensitive and intrusive nature of questions. They had expected the midwife or CFHN to focus more on the health of their unborn or new baby. This is also reported by Hegarty et al. [30] in the UK who indicated that women found the emphasis on them was ‘peculiar’ and they questioned whether the nurse was there for them or the baby. Some of the participants in our study suggested that the real intention of the assessment was somehow hidden from them. It is noteworthy that women expressed that they may have been misled about the intention of services, as in other studies of nurses’ practice, nurses themselves report the covert strategies that they use to gain entry to conduct a home visit [25,31,32]. Shepherd [32] for example, describeds nurses’ acknowledgement that much of the work they do with mothers is hidden ‘behind the scales’ and that the manifest work of weighing the baby is a safe and acceptable way to gain entry to the home.

Our findings suggest that the extent and nature of the questions to be asked is not adequately explained to women prior to the visits where assessment is conducted. Participants described they were not well prepared for certain questions and they would have liked more information about the content of the visit prior to the appointment and at the start of the appointment. Specifically, women did not expect the questions about domestic violence or childhood sexual abuse and one woman compared the routine domestic violence screening to other mandatory security procedures such as going through a customs check at the airport. In a recent Australian study, Rowe et al. [29] asked women about their expectations of the health service in pregnancy and after birth. Women emphasised that they would want to know in advance the type of sensitive questions that they would be asked and they believed that the questions should only be asked by a trained professional who the woman had a relationship with [29]. Similarly, Cowley et al. [18] warn that if women are completely unprepared for this type of questioning it may influence disclosure and women may deny such problems [33].

Some women found aspects of the psychosocial assessment process intrusive such as disclosing past history of child sexual abuse, domestic violence or previous mental health concerns. Women reported discomfort particularly when they were asked to revisit past trauma or felt they were repeating their story and at times feeling they were not prepared to discuss these personal issues. These topic areas such as domestic violence and mental health issues were particularly sensitive for women. Other researchers Raymond et al. and Palmer et al. [34,35] support our findings describing the emotional distress women experienced, at times crying, when asked personal questions during assessment, including domestic violence screening. Phillips et al. [12] p.369 describes a similar finding in substance use disclosure in pregnancy where women described repeating information to health professionals as a ‘pain in the bum’. Other studies have reported women’s discomfort at being asked to open up and discuss sensitive information often not knowing the purpose and how it was to be used [19,33].

Women in this study also experienced emotional distress when responding to other questions that are part of the routine obstetric history in the antenatal booking visit and not categorised as ‘the psychosocial assessment’. Questions relating to previous pregnancies including miscarriage, termination of pregnancy or stillbirth provoked distress in some women. Studies such as Armstrong [36] have shown when women have a previous perinatal loss they experience a mixture of hope and fear of the subsequent pregnancies and most likely experience anxiety and/or guilt [37]. In response, some midwives were observed being particularly sensitive to women who reported pregnancy loss, however there were also instances where midwives and CFHN did not ‘tune in’ to women’s distress [19]. Gilbert [38] emphasises that whilst retelling the story of an event such as stillbirth or miscarriage is therapeutic, it requires trained and highly skilled clinicians who have an understanding of how to facilitate discussions with women around loss and how to respond to such disclosures [39].

Most importantly, and not surprisingly, women’s perception of the style and approach of the midwife/CFHN was the key factor that influenced her experience. Women felt more relaxed and comfortable if they perceived the midwife/CFHN was warm and empathetic. Warmth and empathy were demonstrated through verbal and non-verbal communication. Women from non-English speaking backgrounds emphasised that non-verbal communication such as smiling assisted them to feel calm and facilitated their willingness to engage with the midwife/CFHN. The quality of the communication between the woman and the midwife/CFHN influences whether the midwife or nurse will be able to form an early or beginning relationship with the woman. As Porr et al. [40] describe if a woman thinks nurses consider her needs to be a priority, and if they put effort into getting to know her, this conveys a genuine message that the nurse or midwife cares and that they are there to support the woman. Together with ensuring privacy [12], a non judgemental and empathetic approach in turn facilitates the start of a relationship [41]. If communication is poor, including in accurate recording of events, such as a positive response to domestic violence as illustrated in exemplar (Table 3), Hunter et al. [41] note this may result in sub-standard care and dissatisfaction on the part of women [41].

Most women who were observed in the home context appeared to be comfortable when being asked to provide sensitive information, including the psychosocial assessment and depression screening. However, this appears to differ from previous research. For example, in the UK-based study by Shakespeare et al. [19], women indicated that to ensure privacy and to offer adequate time and a more relaxed approach, it was more appropriate for assessment and screening to be conducted in the home environment, rather than in the baby health clinics [19]. However, some women in this study described that when the visit took place in their home they felt as though they were being watched or monitored. Women found it intrusive when the home visit included assessment of the environment for example, when the nurse commented on the need for safety guards for the stairs. In contrast it appeared the clinic context was less intrusive than the home setting. Although most women who received a home visit appeared to be amenable to allowing the nurse into the home, some did report that they found, at times, the nurse’s approach was inappropriate, rude and or intrusive. This suggests that some women experience the home visit as a form of surveillance and as Wilson found, the idea of a nurse looking around their home can be objectionable [31]. In this situation where a woman may feel that she is being watched and monitored, she is likely to exert a level of control by stopping the service and not attending further sessions, which did occur in this study.

### Implications for practice

These findings demonstrate that a woman’s experience of assessment may be directly impacted upon by the midwife or nurses' approach. The development of a reciprocal trusting relationship with women and families is crucial. The fact that women allow the midwife or nurse into their homes denotes a high level of trust in these universal health services. If this trust is respected and developed by the midwife/nurse, then women are more likely to be open and trust the nurse further [42] and where there is trust women are more likely to disclose their experience [15,18,42-44]. Therefore, it is important for clinicians engaging in this process to build a positive relationship with the woman, always remaining aware that women are wary of criticism, interference or surveillance [45].

The findings related to women’s experience emphasise the need for ongoing supervision and training for midwife/CFHN focusing on skills in building good relationships with women. These skills are needed even in the first encounter, so women feel cared for and supported by the midwife/CFHN. The impact of the relationship and handling a woman’s disclosure in a sensitive manner is more likely to lead women to feel empowered and may be more likely to lead to them seeking help [46,47].

### Limitations

There are a number of limitations to the study. First the study was conducted in only two sites and these differed in terms of length of the interview, at what point in time they occurred, and whether psychosocial assessment was conducted in structured formal ways, or more conversationally [20]. As an in depth ethnographic study, the sample size of 34 women is appropriate, however a third of these women were not available for observations following birth. Most of the women who agreed to participate were well educated with 90% holding tertiary qualifications and therefore may not adequately reflect the experiences of women who have lower levels of education. All participants (women and midwife/CFHN) were aware of the intent of the research, and it is possible that when participants’ are observed they may alter their actions and reactions to present a more ideal performance as mothers and professionals. The potential for social desirability under observation was mitigated by including follow-up interviews with women following each observation, by a researcher who was known to the women.

## Conclusions

This study describes women’s experience of psychosocial assessment and depression screening, revealing what is helpful and the factors that lead to discomfort. Women mostly felt unprepared for the sensitive questions. There were also questions, not viewed by professionals as part of the psychosocial assessment that can cause distress. Some women who disclosed experiences such as loss of a baby or history of child sexual abuse found having to retell their story distressing and would have preferred that the midwife/CFHN referred to previous medical records to source this information. It was important for women to feel supported when they disclosed negative past experiences and personal information and be responded to with sensitivity. Women felt strongly that midwife/CFHN should collaborate around how to record the information they provide in these sensitive interviews.

## Abbreviations

CFHN: Child and Family Health Nurse; SFE: Supporting families early package; NSW: New South Wales; MR: Mellanie Rollans; DG: Discussion groups; W: Woman; FN: Field note.

## Competing interests

There were no competing interests in this study, financial, institutional or otherwise.

## Authors’ contributions

MR: Carried out the data collection, participated in the data analysis and the drafting of this manuscript. VS: Assisted in the design of the study, participated in the data analysis and the drafting of this manuscript. LK: Assisted in the design of the study, participated in the data analysis and the drafting of this manuscript. TC: Assisted in the design of the study, participated in the data analysis and the drafting of this manuscript. All authors read and approved the final manuscript.

## Pre-publication history

The pre-publication history for this paper can be accessed here:

http://www.biomedcentral.com/1472-6874/13/18/prepub
